# Adiabatic two-step photoexcitation effects in intermediate-band solar cells with quantum dot-in-well structure

**DOI:** 10.1038/s41598-019-44335-8

**Published:** 2019-05-27

**Authors:** Shigeo Asahi, Toshiyuki Kaizu, Takashi Kita

**Affiliations:** Department of Electrical and Electronic Engineering, Graduate School of Engineering, Kobe University, Kobe, Japan

**Keywords:** Solar cells, Quantum optics

## Abstract

We studied the dynamics of electrons generated by two-step photoexcitation in an intermediate-band solar cell (IBSC) comprising InAs/GaAs/Al_0.3_Ga_0.7_As dot-in-well (DWELL) structure using time-resolved photocurrent (TRPC) measurement. The examined IBSC exhibited considerably slower photocurrent decay than a conventional InAs/GaAs quantum dot IBSC, which is due to the extraordinarily long-lived electrons in the DWELL. In order to retrieve the electron lifetime from the decay profile, we developed a model reproducing the observed decay and performed parameter fitting. The fitting results indicate that the electron lifetime in the DWELL is approximately 30 μs. In the two-colour excitation TRPC measurement, we found that an additional infrared (IR) light accelerates the photocurrent decay while the photocurrent increases by approximately 3%, because the additional IR light causes two-step photoexcitation of electrons in the DWELLs towards the conduction band. Furthermore, we demonstrated that the open-circuit voltage increases with increasing of the contribution of the second IR excitation process.

## Introduction

For a high-potential candidate of the next-generation solar cells (SCs) with a high conversion efficiency, the intermediate-band solar cells (IBSCs) have drawn significant attention since the concept of IBSC was first proposed in 1997^1^. As compared to the conventional single junction SCs, whose conversion efficiency is restricted by the so-called Shockley–Queisser limit^2^, the IBSC enables to dramatically reduce the transmission loss and is expected to break through the efficiency limit. For example, when an intermediate band (IB) exists in the bandgap, sub-bandgap photons can be absorbed by way of the IB. This adiabatic photo-excitation process increases photocurrent without decreasing the output voltage. Two-step photoexcitation is accomplished by two sequential optical excitations: an initial interband excitation of electrons from the valence band (VB) to the IB and following intraband excitation from the IB to the conduction band (CB). Theoretical calculation of the conversion efficiency based on the ideal detailed balance model predicts 47% under one sun and 63% under the maximum concentration of 46,200 suns^1,3^. In order to realise the IBSC attaining such a high conversion efficiency, efficient two-step photoexcitation is essential^3,4^. In particular, sufficient intraband (intersubband) excitation from the IB to the CB is crucial. Thus far, two-step photoexcitation phenomenon has been experimentally demonstrated at low temperature^5–14^ and even at room temperature^15–21^. However, two-step photoexcitation at room temperature becomes weak because electrons in the IB tend to thermally escape into the CB in case the potential barrier between the IB and the CB is low^13,22–25^. Thermal electron escape pushes down quasi-Fermi level of electrons in the CB, and, therefore, lowers the output voltage. To study pure two-step photoexcitation phenomenon, many experiments have been occasionally performed at low temperature^9–11,22,26^ when thermal electron escape is effectively suppressed. Detailed physics of two-step photoexcitation in InAs/GaAs quantum dot (QD) IBSCs have been clarified^6,7,9–11^. However, even at low temperature, observed two-step photocurrent is still very low. Generally, photogenerated electrons in the IB have a short lifetime of few nanoseconds due to quick recombination with holes before being excited by photons in the two-step photoexcitation process. To extend the lifetime of electrons in the IB, quantum dot superlattices^12–14^ and type II quantum dots^27–30^ have been proposed as possible solutions. Recently, we demonstrated that an IBSC including InAs/GaAs/Al_0.3_Ga_0.7_As dot-in-well (DWELL) structure sufficiently suppresses thermal electron escape at room temperature and attains an extremely long-lived electrons in the DWELL because photogenerated holes are thermally pumped out^16^. This SC is called DWELL-IBSC which achieves efficient two-step photocurrent generation even at room temperature. The external quantum efficiency spectrum of DWELL-IBSC obviously show an enhancement caused by the two-step photocurrent above a threshold wavelength of 930 nm corresponding to the fundamental absorption edge of the InAs wetting layer^16^. In addition, we have investigated the detailed two-step photocurrent generation as functions of the excitation power in interband and intersubband transitions. The two-step photocurrent exhibited saturation when the interband excitation power increased, and the saturation level depended on the intersubband excitation power^19^. We proposed a model to interpret the observed two-step photocurrent and discussed the dynamics of electrons generated in the DWELL. Besides, the simulation results suggested that the electron lifetime in the DWELL has been estimated to be in a range from 400 μs to 2 ms^19^, which is extremely long as compared to the value of conventional InAs/GaAs QDs^31^. In this study, we performed time-resolved photocurrent (TRPC) measurement to directly investigate the electron dynamics with the long lifetime in the DWELL-IBSC. We confirmed an extremely slow response of the photocurrent decay in the DWELL-IBSC. The additional infrared (IR) light irradiation in the two-colour photoexcitation process dramatically accelerates the TRPC decay and recovers the open-circuit voltage.

## Results

**Figure 1:**
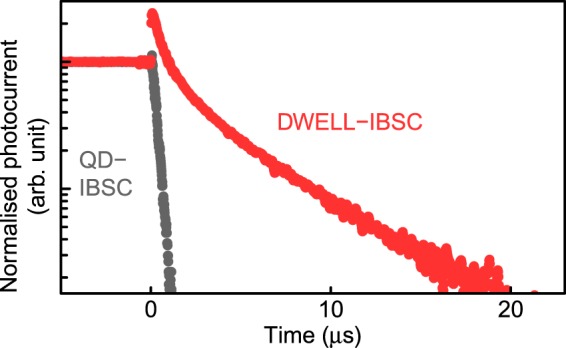
Normalised photocurrent decay profiles for the DWELL-IBSC (red circles) and reference QD-IBSC (black circles) at 300 K.

Figure 1 shows the typical TRPC decay profiles for the DWELL-IBSC and the reference QD-IBSC. Each excitation power density was 3.7 W/cm^2^ for the DWELL-IBSC and 0.21 W/cm^2^ for the reference QD-IBSC, respectively. We detected spike-like signals in the both decay profiles for the DWELL-IBSC and QD-IBSC where the amplitude of the spikes was 0.18 mA/cm^2^. These signal arises from an impedance mismatch between the SCs and the current amplifier. As the spike signal appeared at the very initial stage that we did not focus at, we neglected it in our analysis. For the reference QD-IBSC, the photocurrent rapidly decreases and exhibits a single exponentially decaying curve just after stopping the excitation. The photogenerated electrons in the intermediate states are promptly extracted by thermal escape process because of the relatively shallow confinement energy at room temperature. The estimated decay time is approximately 250 ns, which is predominantly determined by the RC time constant. The estimated junction capacitance of QD-IBSC is 4 nF^32^, and the RC time constant of the SC is estimated to be 200 ns. Conversely, the DWELL-IBSC exhibits a slow, stretched-exponential decay profile. The potential barrier of Al_0.3_Ga_0.7_As is high enough to suppress thermal electron escape even at room temperature, though photogenerated holes are thermally pumped towards the *p*-electrode due to the relatively low barrier height. That causes electron–hole separation, and, therefore, the electron lifetime in the DWELLs is extended. This electron–hole separation was also confirmed by the temperature dependence of the photoluminescence intensity of the DWELL-IBSC^19^. Despite the strong confinement of electrons, they gradually escape in the thermal process, resulting in a very slow and stretched-exponential decay profile. Thus, we directly observed the extraordinarily long-lived electrons in the DWELLs by using the TRPC measurement technique.

**Figure 2:**
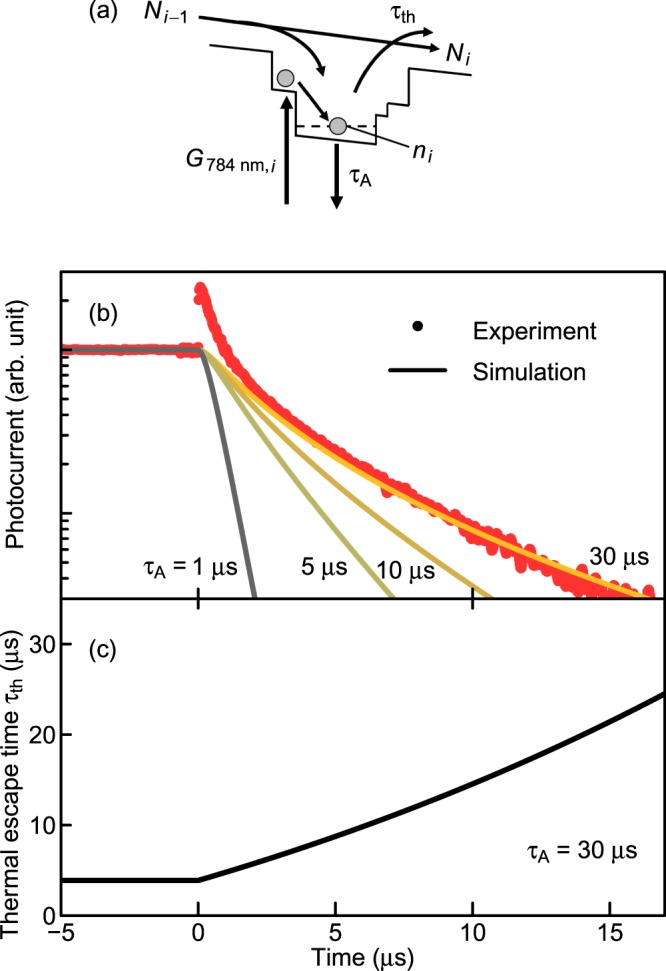
Calculated result of TRPC decay of DWELL-IBSC. (**a**) Schematic numerical model of photocurrent decay profile in the DWELL-IBSC. (**b**) Calculated results of photocurrent decay (solid line). The red circles indicate the observed decay curve for the DWELL-IBSC shown in Fig. 1. (**c**) Calculated thermal escape *τ*_th_. The annihilation time used in the calculation is 30 μs.

In order to retrieve the electron lifetime from the decay profile, we propose a model reproducing the decay curve. In this model, we neglected dynamics contributed by holes because of quick thermal escape from the DWELL. Figure 2(a) illustrates the model of the CB lineup. The rate equation representing the electron density per unit area in the *i*th DWELL, *n*_*i*_, is given by

1 $$\frac{{\rm{d}}{n}_{i}}{{\rm{d}}t}=s{G}_{784\mathrm{nm},i}-\frac{{n}_{i}}{{\tau }_{{\rm{A}}}}-\frac{{n}_{i}}{{\tau }_{{\rm{th}}}}+\gamma {N}_{i-1},$$

where *G*_784 nm,*i*_ is the interband photocarrier generation rate in the *i*th DWELL per unit area, *τ*_A_ and *τ*_th_ are the annihilation time and thermal escape time in the DWELL, respectively. *N*_*i*−1_ is the generated areal density of electrons supplied from the (*i*−1)th DWELL per unit time and is defined as follows:

2 $${{N}}_{i-1}=\frac{{n}_{i-1}}{{\tau }_{{\rm{th}}}}+(1-\gamma ){{N}}_{i-2}.$$

γ is the fraction of electrons trapped into the *i*th DWELL. *s* is the fraction of long-lived electrons in the DWELL owing to the electron–hole separation. Part of electrons in the DWELL quickly recombines with holes within few nanoseconds, and the remaining electrons slowly decay in the thermal escape process. Thus, *sG*_784 nm,*i*_ corresponds to the generation rate of long-lived electrons. *sG*_784 nm,*i*_ becomes zero after stopping the irradiation of the LD (*t* > 0). The second and third terms on the right-hand side of Eq. (1) represent the annihilation rate in the DEWLL and the thermal escape rate of electrons, respectively. The electrons separated from holes can be alive much longer than the radiative recombination time. The annihilation time of electrons is, therefore, mainly determined by the Shockley–Read–Hall recombination and is expected to extend the lifetime into a temporal region of microseconds ~ milliseconds. We assumed that thermally extracted electrons immediately reach the next DWELL because the estimated drifting time through the 50-nm-thick Al_0.3_Ga_0.7_As barrier is less than one picosecond, which is negligible as compared to the temporal scale of interest. For simplicity, we neglected band bending due to electron accumulation in DWELLs and nonlinear effect such as Auger effect. In addition, we used same *s* and γ values (0.01 and 0.08) for all DWELL-layers in Eq. (1). It should be noted that Eq. (1) presents an electron balance model in one DWELL-layer, and the unit of *n*_i_ is m^−2^. *G*_784 nm,*i*_ is obtained by using the Beer–Lambert law as follows

3 $${G}_{784\mathrm{nm},{\rm{i}}}={P}_{784\mathrm{nm},i}\{1-\exp [-(1-{f}_{{\rm{well}},i}){\alpha }_{{\rm{well}}}{d}_{{\rm{well}}}]\},$$

where *P*_784 nm,*i*_ is the incident photon flux of the 784-nm LD light source at the *i*th DWELL, *f*_well,*i*_ is the electron occupation factor of the GaAs QW states in the *i*th DWELL, 𝛼_well_ is the interband absorption coefficient of the QW, and the QW thickness *d*_well_ is 16 nm. *P*_784 nm,*i*_ is calculated from the excitation power density considering the reflectivity of the SC surface and the photon energy. We considered that the 784-nm LD excites the fundamental states of the GaAs QW. The state filling effect is also taken into account in Eq. (3). We used 3,000 cm^−1^ for 𝛼_well_^33^. The quasi-Fermi level of electrons in the DWELL is described using the Fermi–Dirac distribution

4 $${{f}}_{{\rm{well}},i}=\frac{1}{1+\exp [({E}_{{\rm{well}}}-{E}_{{\rm{f}},i})/{kT}]}\approx \exp (\frac{{E}_{{\rm{f}},i}-{E}_{{\rm{well}}}}{kT}),$$

where *E*_well_ is the electron energy level of the fundamental state of the GaAs QW, *k* is the Boltzmann constant, *T* is the temperature (300 K), and *E*_f,*i*_ is the quasi-Fermi level of the electrons of the *i*th DWELL. Here, we used the Boltzmann approximation in Eq. (4) because *E*_f,*i*_ is sufficiently lower than *E*_well_. *E*_f,*i*_ can be expressed as follows

5 $${{E}}_{{\rm{f}},i}={{E}}_{{\rm{equil}},i}+{k}T\,\mathrm{ln}(\frac{{n}_{i}}{{n}_{{\rm{intr}},i}}),$$

where *E*_equil,*i*_ is the Fermi-level in the state of thermal equilibrium and *n*_intr,*i*_ is the intrinsic areal electron density in the *i*th DWELL. To calculate *τ*_th_, we used the thermionic emission model of QWs described as^34,35^

6 $${\tau }_{{\rm{th}}}={d}_{{\rm{well}}}\sqrt{\frac{2\pi {m}^{\ast }}{{kT}}}\exp (\frac{{{E}}_{{\rm{AlGaAs}}}-{{E}}_{{\rm{f}},i}}{kT}),$$

where m^*^ is the effective electron mass and *E*_AlGaAs_ is the CB edge of Al_0.3_Ga_0.7_As. We used 6.1 × 10^−32^ kg for m^*^. We substituted Eqs (2–6) into Eq. (1), and, then, numerically calculated *n*_*i*_ in *i*th DWELL. Thereby, the calculated short-circuit current density, *J*_sc_, was obtained from following equations

7 $${{J}}_{{\rm{sc}}}=q{{N}}_{10},$$

where *q* is the elementary charge.

Solid lines in Fig. 2(b) indicate numerically calculated TRPC decay profiles for the DWELL-IBSC at different *τ*_A_’s. With the increase of *τ*_A_, the calculated decay approaches the experimental results. According to a curve fitting to reproduce the observed decay curve, *τ*_A_ is estimated to be 30 μs. Figure 2(c) shows the calculated *τ*_th_ at *τ*_A_ of 30 μs. *τ*_th_ is a few microseconds during the excitation and exhibits a monotonic increase after stopping the excitation. Since *τ*_th_ is smaller than *τ*_A_ at time < 20 μs, the decay of DWELL-IBSC shown in Fig. (1) is predominantly determined by *τ*_th_. The estimated *τ*_A_ becomes smaller than the value obtained by a systematic excitation power dependence of the two-step photocurrent, which is in the range from 400 μs to 2 ms^19^. *τ*_A_ evaluated by the TRPC measurements in this work is believe to be more precise rather than the value predicted by the analysis of carrfier dynamics based on data measured at the steady state condition.

**Figure 3:**
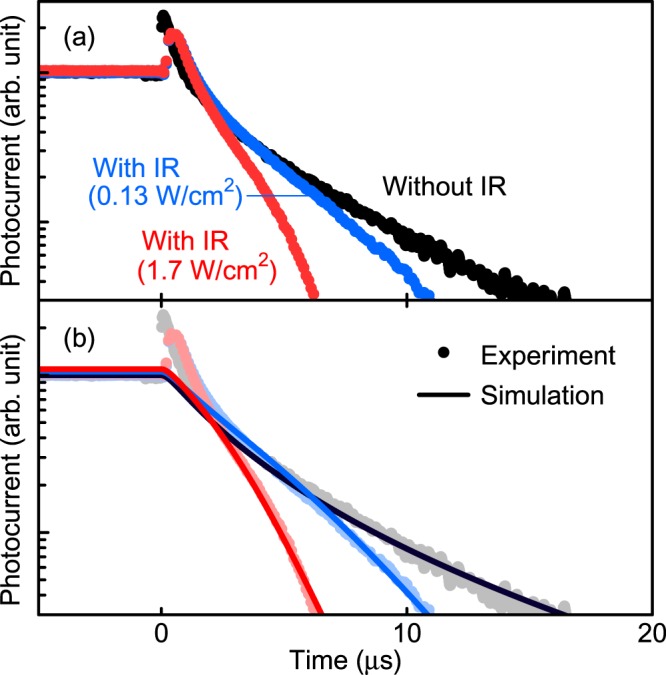
Photocurrent decay profiles with additional IR light. (**a**) Photocurrent decay profiles for the DWELL-IBSC with different power densities of the additional IR light. (**b**) Calculated results of the photocurrent decay profiles with the additional IR light.

Subsequently, we performed two-colour excitation TRPC measurements for the DWELL-IBSC to investigate the temporal behaviour when irradiated by IR light at 300 K. Figure 3(a) shows the results. In this experiment, we used a supercontinuum white laser light passing through a long pass filter transmitting IR light above 1,250 nm. The fundamental transition energy of QDs in the DWELL-IBSC is 1.05 eV (1,180 nm) at 300 K so that the additional IR light only excites the states in the intraband of the DWELLs^16^. We confirmed that the observed photocurrent obeys linear relationship with the IR photon density, indicating non-linear phenomenon such as two-phonon absorption does not occur in this measurement. The first interband excitation light used and its power density were the same as those in the experiment shown in Fig. 1. It is noted that the photocurrent profiles shown in Fig. 3(a) are un-normalised, original data. We found that the additional IR light accelerates the photocurrent decay while the photocurrent increases by ~3% at the IR power density of 1.7 W/cm^2^. The additional IR light causes two-step photoexcitation of electrons in the DWELLs, resulting in this fast decay profile. In order to interpret the decay profile under the irradiation of the additional IR light, we took into account the term of two-step photocurrent generation rate, *G*_IR,*i*_, in Eq. (1) as follows

8 $$\frac{{\rm{d}}{n}_{i}}{{\rm{d}}t}=s{G}_{784\mathrm{nm},i}-\frac{{n}_{i}}{{\tau }_{{\rm{r}}}}-\frac{{n}_{i}}{{\tau }_{{\rm{th}}}}+\gamma {N}_{i-1}-{G}_{{\rm{IR}},i}.$$

**Figure 4:**
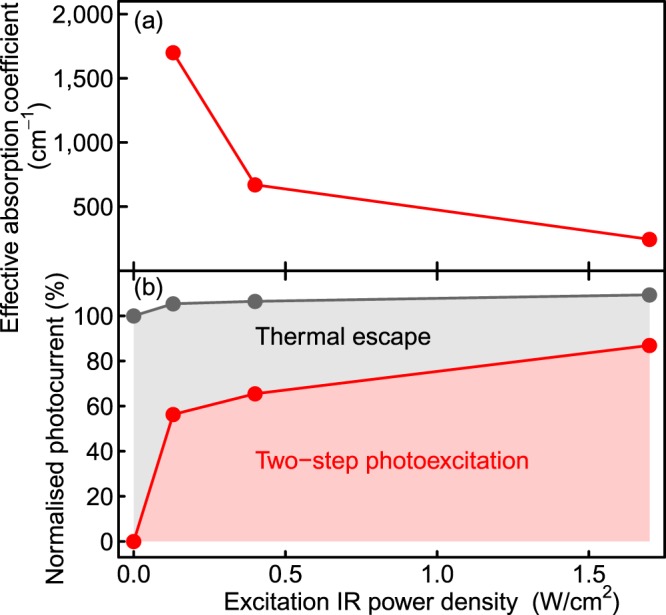
Retrieved fitting values from the simulation. (**a**) Intraband absorption coefficient of intraband excitation for InAs QD as a function of excitation IR power density. (**b**) Calculated cause of the photocurrent which is normalised by the photocurrent without IR excitation.

*G*_IR,*i*_ can be expressed as

9 $${G}_{{\rm{IR}},i}={P}_{{\rm{IR}},i}[1-\exp (-{f}_{{\rm{dot}},i}{\alpha }_{{\rm{dot}}}{d}_{{\rm{dot}}})],$$

where *P*_IR,*i*_ is the incident photon flux of the IR light, *f*_dot,*i*_ is the *effective* occupation factor at the QD states in the *i*th DWELL, 𝛼_dot_ is the *effective* absorption coefficient of InAs QD, and the InAs QD layer thickness *d*_dot_ is 4 nm. We considered that the intraband transition does not occur in the QW states and only occurs in the QD states, because the optical dipole transition for the intraband transition in quantum structures is only allowed for the component polarized parallel to the confined direction^36^. Here, it is noted that, in our model as illustrated in Fig. 2(a), we simply assumed a single electron energy level as the initial state in InAs QD. However, according to the PL measurements^16^, at least three quantized states play the role of the initial state. Therefore, *f*_dot,*i*_ is the *effective* occupation factor for the initial state containing several quantized states in QDs, and α_dot_ is the *effective* absorption coefficient for the initial state. We assumed that two-step photoexcitation occurs at the fundamental and excited states of QDs. *f*_dot,*i*_ can be calculated as

10 $${f}_{{\rm{dot}},i}=\frac{1}{1+\exp [({E}_{{\rm{dot}}}-{E}_{{\rm{f}},i})/{kT}]},$$

where *E*_dot_ is the electron energy level of InAs QD. We assume that the states of GaAs QW and InAs QD are thermally coupled and used the same value for *E*_f,i_ in Eqs (4 and 10). Figure 3(b) summarises the calculation results. The calculation results agree well with the experimental data and reproduce that the additional IR light accelerates the photocurrent decay. We found that the intraband effective absorption coefficient of InAs QD 𝛼_dot_ changes as a function of excitation intensity. The best fit 𝛼_dot_ values are summarised in Fig. 4(a). 𝛼_dot_ decreases with excitation IR power density. As the additional IR light increases, the quasi-Fermi level of DWELL falls, and the probability of intraband excitation decreases, leading to the decrease in 𝛼_dot_ with the IR power density. The evaluated intraband absorption coefficient is in the range of 200–1,800 cm^−1^, which is comparable to the reported values^7,37^. By distinguishing the current of thermal escape and two-step photoexcitation, we evaluated the amount of the photocurrent caused by the additional IR light illumination. The estimated result is drawn in Fig 4(b). Without the additional IR light, photocurrent is only caused by thermal electron escape. In contrast, when irradiated by the additional IR light, electrons are also extracted by two-step photoexcitation. Two-step photocurrent increases as the power density of the additional IR light increases. Conversely, as electrons are extracted by the additional IR light irradiation, the electron density in the DWELL decreases and the contribution of the thermal electron escape decreases for that. As the detected photocurrent is a sum of the decreased thermally escaped current and the increased two-step photocurrent, the change in the detected photocurrent by the additional IR light is small. When the power density of the additional IR light was 1.7 W/cm^2^, ~80% of the detected photocurrent is caused by the two-step photocarrier generation.

**Figure 5:**
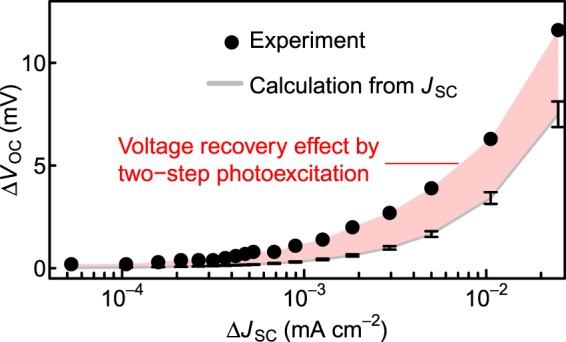
Change in the open-circuit voltage (Δ*V*_OC_) as a function of the change in the short-circuit current (Δ*J*_SC_) under the irradiation of the additional IR light measured at 300 K. The grey line indicates Δ*V*_OC_ estimated from experimentally observed short-circuit current (*J*_SC_) using Eq. (11). The errors represent uncertainty for diode factor *n* of 1.2 ± 0.1, which is determined from the slope of dark Log *J*–*V* curve. The red area is the difference between the experimental observation and the estimation Δ*V*_OC_, interpreted as the voltage recovery by the two-step photoexcitation.

When only irradiated by the 784-nm LD, quasi-Fermi level is single. When the additional IR light pumps electrons from the IB into the CB, quasi-Fermi levels of the IB and the CB start splitting, and, therefore, the adiabatic process of the two-step photoexcitation recovers the output voltage^38^. In order to demonstrate the voltage recovery effect caused by two-step photoexcitation, we investigated short-circuit current, *J*_SC_, and open-circuit voltage, *V*_OC_, under the irradiation of the both sources, the 784-nm LD and the additional IR light. The solid circles in Fig. 5 show the measured Δ*V*_OC_ as a function of Δ*J*_SC_. Here, Δ*J*_SC_ and Δ*V*_OC_ are the changes in *J*_SC_ and *V*_OC_ caused by the additional IR light irradiation, respectively. In this measurement, the power density of 784-nm LD was 3.7 W/cm^2^, and the power density of the additional IR light was in the range from 58 mW/cm^2^ to 11 W/cm^2^. Δ*V*_OC_ dramatically increases with Δ*J*_SC_.

## Discussion

Both the photocurrent increase and the voltage recovery caused by the quasi-Fermi level splitting contribute to Δ*V*_OC_. Δ*J*_SC_ itself increases *V*_OC_. In order to visualise the voltage recovery effect, we compare the experimentally observed Δ*V*_OC_ with Δ*V*_OC_ intrinsically expected to be caused by Δ*J*_SC_. According to the detailed balance model for the ideal single junction SC, the relationship between *J*_SC_ and *V*_OC_ obeys the following equation

11 $${{V}}_{{\rm{oc}}}=\frac{nkT}{q}\,\mathrm{ln}(\frac{{J}_{{\rm{sc}}}}{{J}_{0}}+1),$$

where *n* is diode quality factor, *J*_0_ is saturation current density. First, we substituted measured *V*_OC_ and *J*_SC_ under the irradiation of the 784-nm LD only into Eq. (11) and obtained *J*_0_ of 3.7 × 10^–2^ mA/cm^2^. Here, we used 1.2 ± 0.1 for *n*, which is determined from the slope of dark Log *J*–*V* curve of the DWELL-IBSC. Utilising *J*_0_, we calculated *V*_OC_ by substituting *J*_SC_ measured when irradiated by the IR light into Eq. (11). The calculated results are depicted by the grey solid line in Fig. 5. The experimentally observed Δ*V*_OC_ apparently surpasses the values increased by Δ*J*_SC_, and the difference indicated by the red area in Fig. 5 in between them corresponds to the voltage recovery caused by the quasi-Fermi level splitting. It demonstrates the principle concept of the two-step photocarrier generation in IBSC in which total photocurrent increases without decreasing of the photovoltage.

## Methods

### Solar cell fabrication

An Al_0.3_Ga_0.7_As *p–i–n* SC device structure was fabricated on an *n*^+^-type GaAs (001) substrate by using solid-source molecular beam epitaxy. An *n*-Al_0.3_Ga_0.7_As (700 nm)/*n*^+^-Al_0.3_Ga_0.7_As (150 nm) layer was deposited on a 400-nm-thick *n*^+^-GaAs buffer layer. Subsequently, a 1400-nm-thick *i*-layer including a DWELL structure was deposited. The *i*-layer consisted of Al_0.3_Ga_0.7_As (540 nm), ten periods of GaAs (10 nm)/InAs QDs (two monolayers)/GaAs (6 nm)/Al_0.3_Ga_0.7_As (50 nm), and Al_0.3_Ga_0.7_As (200 nm) layers. Finally, a 150-nm-thick *p*-Al_0.3_Ga_0.7_As emitter layer was grown. The detailed SC structure and its band diagram have been reported in our previous publications^16,19^. In addition, we also fabricated a conventional InAs/GaAs QD-IBSC with the same *i*-layer thickness and ten QD layers as a reference IBSC.

### TRPC measurement

In order to elucidate the dynamics of photogenerated electrons in the DWELLs, we performed TRPC measurement for the two IBSCs. Two laser diodes (LDs) with different wavelengths were used for exciting the SCs; a 784-nm LD was used for the DWELL-IBSC, and a 940-nm LD was used for the reference QD-IBSC. Both LDs excited the intermediate state^16^. The LD output power was directly modulated by a function generator that outputs a square-wave signal with a frequency of 100 Hz and a duty ratio of 50%. We detected short-circuit photocurrent by using a current amplifier and a digital oscilloscope triggered by the function generator. The temporal response time of the detection system was ~20 ns, which is sufficiently fast to investigate the electron dynamics in the SCs. All measurements were conducted at 300 K.

### Theoretical simulation

We performed calculations using Visual Studio Community 2017. The programming language used was Visual C++.

## Data Availability

The data that support the findings of this study are available from the corresponding author upon request.

## Code Availability

The computer code used in this study are available from the corresponding author upon request.
